# Media narratives of kindness − a critique

**DOI:** 10.1177/1329878X20953278

**Published:** 2020-11

**Authors:** Mohan Dutta, Phoebe Elers

**Affiliations:** Massey University, New Zealand

**Keywords:** COVID -19 and poverty, COVID -19 communication, culture-centred approach, media communication and COVID -19, narratives of kindness

## Abstract

Since the coronavirus outbreak, various media outlets across the globe have disseminated and promoted stories of kindness as registers for COVID-19 response. These narratives of kindness appeal to the human capacity to do good, inviting the public to ‘make a difference’ by performing altruistic acts of helping those who are less fortunate or in a time of need. However, there is a dark side to these narratives, one that does not serve those who are precarious, underprivileged or marginalised, by obfuscating and erasing necessary conversations on the transformative policies and infrastructure changes needed to address fundamental structural issues underlying the deep inequalities we inhabit in a COVID world.

Since the coronavirus outbreak, various media outlets across the globe have disseminated and promoted stories of kindness as registers for COVID-19 response. In this essay, we critically interrogate media narratives around kindness circulated in mainstream discourse in India, Singapore and New Zealand. Indian news articles featured headlines such as ‘Kindness is our hope in the post-COVID-19 world’ (Sen, 2020: para. 1) and ‘India coronavirus: How police won hearts with cakes, songs and sacrifice’ (Pandey, 2020: para. 1), while the #sparkthejoy social media campaign encouraged people to do an ‘act of good’ (Borah, 2020). Similar news articles featured in Singapore, with statements such as ‘The astounding, unprecedented outpouring of concentrated kindness does indeed show us something new about our city-state’ (Heng, 2020: para. 31) and the reporting on the ‘Raya Kindness Packs’ (Han, 2020) that were given to town council cleaners in appreciation. In New Zealand, news outlets reported on teddy bears being displayed in household windows for children (Beck, 2020) and neon signs flashing ‘Be kind, stay calm’ on motorways (Roy, 2020). These narratives of kindness appeal to the human capacity to do good, inviting the public to ‘make a difference’ by performing altruistic acts of helping those who are less fortunate or in a time of need. However, there is a dark side to these narratives, one that does not serve those who are precarious, underprivileged or marginalised, by obfuscating and erasing necessary conversations on the transformative policies and infrastructure changes needed to address fundamental structural issues underlying the deep inequalities we inhabit in a COVID world. For instance, the narrative of kindness was mobilised to channel public concerns around the outbreaks in dormitories housing low-wage migrant workers towards participation in state-controlled initiatives of serving workers while simultaneously being deployed to silence activists and workers raising critical concerns about the poor treatment of the workers (Wham, 2020).

Coronavirus travels across routes of global movements of labour and human bodies, but it also travels unequally, such that it tends to leave its mark on sites and spaces that are left most vulnerable with poorer infrastructures. A key element of the neoliberal transformation is the active creation of ‘displacement’ (Harvey, 2002) or ‘processes of expulsion’ (Sassen, 2014) where communities in the global margins are actively worked on in order to expel them from their everyday spaces of life and livelihood. This is marked by the proletarianisation of labour, in which migrants are located at the material margins of mainstream societies (Dutta, 2011), causing communities at the global margins to be expelled from their spaces of livelihood, into ever-floating networks of capitalist extraction (Dutta, 2012, 2017a; Pal and Dutta, 2013). This often takes place through the language of ‘mobility’ and of ‘creating opportunities’, with little attention to how movements of those located at the margins are often constituted and necessitated by structural disenfranchisement (Dutta and Kaur-Gill, 2018). The neoliberalisation of economies produces immobilities, whereby those who are most vulnerable struggle to gain access to meaningful migration, while working as temporary and expendable labour (Dutta and Kaur-Gill, 2018). Displacement has been witnessed in various contexts, including among low-income Bangladeshi and migrant workers in Singapore (Dutta, 2017b) and rural populations in India (Pal and Dutta, 2013) and China (Sun and Dutta, 2016). In New Zealand, many *Māori* (Indigenous peoples) moved to urban sites in the 1950s (Harris, 2004), and recent interviews in these low-income areas have identified struggles in affording healthy food, healthcare and housing (Elers et al., 2020).

In some contexts, the recent coronavirus outbreak has made the fundamental disjuncture between the narrative of ‘mobility’ and the actual lived experiences of being precarious visible. In Singapore, low-income migrant workers are considered a ‘hidden backbone’, with their dormitories located on the outskirts of the city (Leung, 2020) and their voices being unheard in mainstream discourses (Dutta, 2020c). Once considered a ‘coronavirus success story’, the recent outbreak of coronavirus in areas where these workers reside drew media attention to their circumstances and highlighted what can happen when communities who are situated in the margins are invisibilised during a health crisis (Leung, 2020). Interviews conducted in April 2020 found that preventive measures for coronavirus can be impossible in these spaces, such as proper hygiene and social distancing, as migrant dormitories are frequently unclean and overcrowded, with between 15 and 20 workers in a room (Dutta, 2020b). Similar concerns have been raised in Indian media outlets with headlines such as ‘Social distancing is a privilege of the middle class. For India’s slum dwellers, it will be impossible’ (Sur and Mitra, 2020: para. 1). In addition, millions of migrant workers in India have already travelled from cities to rural villages (Mahbubani, 2020), posing the risk of further spreading the virus to areas with underdeveloped healthcare infrastructures. To date, New Zealand has a relatively low number of coronavirus cases, but the overcrowding in some low-income Pasifika households in suburban Auckland (Elers et al., 2020) leaves these communities more vulnerable. Furthermore, residents in a low-income area in Palmerston North have identified challenges in accessing healthcare and health information during the recent coronavirus lockdown (Dutta, 2020a), which has been echoed in ongoing interviews conducted with other low-income groups in New Zealand.

Set against the backdrop of these deep inequalities and forms of marginalisation that are made visible by the coronavirus outbreak, the media narrative of kindness appeals to an individualised form of care. Frequently, this narrative was also urged by health organisations (e.g. Mental Health Foundation, 2020) and Governments, such as the New Zealand Government (2020) which stated ‘Look after others. Kindness is an incredibly powerful way to show we are united against COVID-19’ (para. 1–2) as a way to encourage people to stay socially engaged and support one another in slowing the spread of coronavirus. There have been various initiatives that have assisted people situated at the margins during the coronavirus outbreak in different contexts, such as the delivery of food and soaps (e.g. Heagney and Jacobs, 2020; Heng, 2020) which are needed and are responsive to some immediate problems. However, they are also ‘band aids’, by not addressing structural issues and broader determinants of health, and we must recognise that. The invitation to ‘kindness’, expressed as ‘togetherness’, ‘altruism’ and providing services to the now visible people situated in the margins, may alleviate guilt and unite populations. At the same time, it is a fundamental infrastructure of neoliberalism, because it keeps power structures intact by discouraging critique while simultaneously continuing to erase spaces of articulation from the margins by failing to provide real avenues for their voices. For instance, when the conditions of housing for migrant workers were raised by Cooperative for Assistance and Relief Everywhere (CARE) as well as activists, the authoritarian state in Singapore turned to the kindness narrative to define legitimate citizen action in response to the outbreak as well as to label forms of critical interrogation of the failure of the state as anti-national, against the interest of the state (see Wham, 2020).

An inherent issue is that the show of kindness is likely to disappear when the focus on coronavirus disappears, and, once again, the margins are going to be made invisible and erased, while their day-to-day struggles move out of the policy agenda. Consider, for instance, the ongoing experiences of depression and challenges to mental health experienced by low-wage migrant workers in Singapore. Without adequate communicative infrastructures for raising their voices, low-wage migrant workers are left without avenues for advocating for their mental health concerns. As civil society and public attention have shifted, the ongoing challenges experienced by the workers, including the challenges to mental health, remain unaddressed. In this sense, the narrative of kindness perpetuates erasure, because it does not invite the public to question the fundamental power inequalities that are constituted within societies. Interrogating the neoliberal tropes propped up by calls to kindness, we argue that the struggles for addressing structural equality and for responding to the challenges of poverty and access among those at the margins have to begin by building voice infrastructures and agitating towards communicative equality (see Dutta, 2008, 2016). The discursive framework of foregrounding kindness in New Zealand for instance, supported by material policies of support for those struggling economically, must be sustained and supported by strengthening of unions and communicative infrastructures for collectivisation at the margins. Building communicative equality seeds radical democracies for addressing the health and well-being needs of those at the margins. Our critical interrogation of the hegemonic narrative of kindness suggests that this is a moment to think about the long-term sustainable solutions for the future and the kinds of policy changes that we need within national and global spaces, anchoring these solutions in democratic struggles for voice equality at the margins of our societies.
